# Macrophages: emerging targets for ulcerative colitis

**DOI:** 10.3389/fimmu.2025.1623491

**Published:** 2025-09-15

**Authors:** Siqing Chen, Zhang Qin, Xiaoyuan Lin, Sainan Zhou, Yin Xu, Ying Zhu

**Affiliations:** ^1^ Department of Gastroenterology, The First Hospital of Hunan University of Chinese Medicine, Changsha, Hunan, China; ^2^ The Fourth Hospital of Changsha (Changsha Hospital Affiliated with Hunan Normal University), Changsha, Hunan, China

**Keywords:** ulcerative colitis (UC), macrophage, immune, inflammation, treatment

## Abstract

**Background:**

Ulcerative colitis (UC) is a chronic inflammatory bowel (IBD) disease characterized by a complex pathogenesis and limited treatment options. Macrophages play a key role in the pathophysiology of UC by regulating inflammatory responses and tissue repair processes. Currently, there is no comprehensive summary of macrophage regulatory pathways in UC, either domestically or internationally.

**Objective:**

This review aims to systematically elucidate the role of macrophages in UC and their specific regulatory mechanisms, and to identify potential therapeutic strategies and future research directions.

**Methods:**

A comprehensive literature review was conducted, integrating recent advances from global studies to explore macrophage-related pathways and functional alterations in UC. Special attention was given to studies investigating molecular mechanisms underlying macrophage polarization and function.

**Results:**

Evidence indicates that macrophage dysfunction is a central mechanism in the pathogenesis of UC. Major findings demonstrate that metabolic reprogramming serves as a fundamental pathway inducing phenotypic and functional alterations in macrophages. Additional mechanisms mediating these changes include epigenetic modifications, chemokine-driven recruitment, microbial metabolite induction, autophagy, and apoptosis. Multiple drugs targeting macrophages have shown effectiveness in treating UC.

**Conclusion:**

Targeting macrophage-related pathways represents an effective therapeutic approach for UC. This review provides a theoretical foundation for developing precision treatments focused on macrophage modulation and highlights important new avenues for future research.

## 1 Introduction

Ulcerative colitis (UC), a chronic relapsing-remitting inflammatory bowel disease (IBD), imposes a substantial global health burden with rising incidence in industrialized nations (1). Characterized by diffuse mucosal inflammation extending from the rectum to the colon, UC manifests as bloody diarrhea, abdominal pain, and systemic complications, significantly impairing patients’ quality of life (2).It can be classified into distinct subtypes based on the anatomical extent and severity of mucosal inflammation. Clinically, UC is commonly categorized into proctitis (limited to the rectum), left-sided colitis (extending up to the splenic flexure), and pancolitis (diffuse involvement of the entire colon) (3). These classifications have significant implications for both therapeutic decision-making and long-term prognosis: localized proctitis often responds well to topical therapies, whereas pancolitis typically requires systemic immunosuppression or biologic agents. Furthermore, UC can be stratified by disease activity using validated scoring systems such as the Mayo score or the simple clinical colitis activity index, which integrate parameters including stool frequency, rectal bleeding, systemic symptoms, and endoscopic findings (4, 5). Accurate classification not only guides optimal treatment strategies but also aids in predicting complications such as steroid dependency, hospitalization risk, and the long-term development of colitis-associated cancer (6). Despite advances in anti-inflammatory and immunosuppressive therapies, some patients exhibit inadequate response to conventional treatments, highlighting the unmet need for novel therapeutic strategies targeting the core pathogenic mechanisms (7). The pathophysiology of UC involves complex interactions between genetic predisposition, epithelial barrier dysfunction, dysregulated immune responses, and environmental triggers (8, 9). Within this intricate network, macrophages emerge as pivotal orchestrators of intestinal homeostasis and inflammation (10, 11). As the most abundant population of innate immune cells in the gut mucosa, macrophages demonstrate remarkable functional plasticity, dynamically shifting between pro-inflammatory (M1-like) and tissue-reparative (M2-like) phenotypes in response to microenvironmental signals (12). Emerging evidence suggests that macrophage polarization imbalance - skewed toward pro-inflammatory states during active inflammation and insufficient resolution during remission - constitutes a critical driver of UC pathogenesis (13). This functional duality positions macrophages as both instigators of tissue damage and architects of mucosal healing, creating a therapeutic paradox that demands precise regulatory interventions.

Recent breakthroughs have unveiled multidimensional regulatory mechanisms governing macrophage behavior in UC. Metabolic reprogramming, particularly shifts in glycolysis, oxidative phosphorylation (OXPHOS), amino acid metabolism, and lipid metabolism, has been identified as a master regulator of macrophage polarization (14, 15). Concurrently, epigenetic modifications including DNA methylation, histone acetylation, and non-coding RNA regulation have been shown to establish long-term activation states in gut macrophages (16, 17). Furthermore, the chemokines modulate macrophage functional differentiation through intricate crosstalk with other immune cells (18, 19). Notably, the microbiota and its metabolites engage in bidirectional communication with macrophages via pattern recognition receptors and metabolite-sensing pathways, creating a self-perpetuating cycle of dysbiosis and immune dysregulation (20, 21). Despite these advances, current literature lacks a systematic integration of macrophage-centered regulatory networks in UC. In this review, we aim to bridge this gap by synthesizing recent findings into a cohesive framework of macrophage regulation in UC. We systematically examine five major regulatory axes:1) metabolic reprogramming driving functional polarization, 2) epigenetic modifications establishing activation thresholds, 3) chemokine networks shaping microenvironmental communication, 4) macrophage apoptosis and colitis, and 5) microbiota-macrophage crosstalk maintaining mucosal homeostasis. By critically evaluating preclinical and clinical studies, we highlight promising therapeutic strategies for UC. Finally, we propose future research directions that emphasize multi-dimensional drug formulations to precisely target macrophages for UC.

## 2 Fundamental biological characteristics of macrophages

Macrophages, with their diverse origins, phenotypic plasticity, and functional versatility, serve as central regulators in inflammatory diseases. Understanding their fundamental biology is essential for elucidating their mechanistic roles in pathological processes. As key components of the innate immune system, macrophages not only initiate immune and inflammatory responses against pathogens but also maintain tissue homeostasis and contribute to tissue repair and remodeling (22, 23). Monocytes, the precursors of macrophages, originate from hematopoietic stem cells in the bone marrow. These monocytes circulate in the bloodstream as resident or inflammatory monocytes and, upon migrating to peripheral tissues, differentiate into tissue-resident macrophages under specific microenvironmental cues (24). Notably, most tissue-resident macrophages are embryonically derived (25), originating from the yolk sac, fetal liver, or bone marrow, whereas macrophages recruited to tissues in response to injury or infection arise from bone marrow-derived hematopoietic stem cells later in life (26). Colonic macrophages are primarily found in the colonic tissue and represent one of the largest populations of cells in the colonic microenvironment. They play a significant role in the immune response and inflammatory processes of the colon (27). Peritoneal macrophages, which originate from embryos, are the primary immune cells found in the abdominal cavity and are essential for maintaining immune homeostasis. It is important to note that the gut is part of this abdominal environment. There are distinct differences in the distribution and functional characteristics of peritoneal and colonic macrophages. These differences enable each type of macrophage to carry out unique roles under various physiological and pathological conditions. For instance, intraperitoneal injection of mitomycin C can activate peritoneal macrophages and induce colitis in rats (28). Conversely, the administration of NF-κB inhibitors via intraperitoneal delivery has been shown to improve severe colitis by facilitating the migration of drug-carrying macrophages from the peritoneal cavity to the site of inflammation (29). These indicate that the activation and migration of peritoneal macrophages are also closely related to the occurrence and development of colitis. Macrophages are ubiquitously distributed across all tissues and organs, making them indispensable to the innate immune system (23).

Macrophages are highly plastic cells capable of adopting diverse functional phenotypes in response to microenvironmental signals (30). The classical dichotomy describes two major activation states: classically activated (M1) and alternatively activated (M2) macrophages. M1 macrophages are typically induced by pro-inflammatory cytokines and exhibit potent immunostimulatory and tumoricidal activity, often contributing to tissue damage during chronic inflammation (31–33). In contrast, M2 macrophages — sometimes referred to as “wound-healing” macrophages — promote anti-inflammatory responses, tissue repair, and resistance to parasitic infections (31–33). However, the M1/M2 dichotomy is an oversimplified model (34, 35). Macrophage phenotypes exist along a continuum, with dynamic interconversion between states under specific conditions, finely regulated by metabolic reprogramming, epigenetic modifications and other pathways (36).

Functionally, macrophages play essential roles in both innate immunity and tissue homeostasis. Upon recruitment to inflammatory sites, they migrate into affected tissues, where they phagocytose cellular debris and release bioactive mediators that modulate immune responses, inflammation, and tissue repair (37, 38). Furthermore, macrophages act as antigen-presenting cells, presenting processed antigens to T cells and thereby initiating adaptive immune responses (39). They also secrete chemokines that regulate the activation and trafficking of other immune cell populations, reinforcing the crosstalk between innate and adaptive immunity (40).

**Figure 1:**
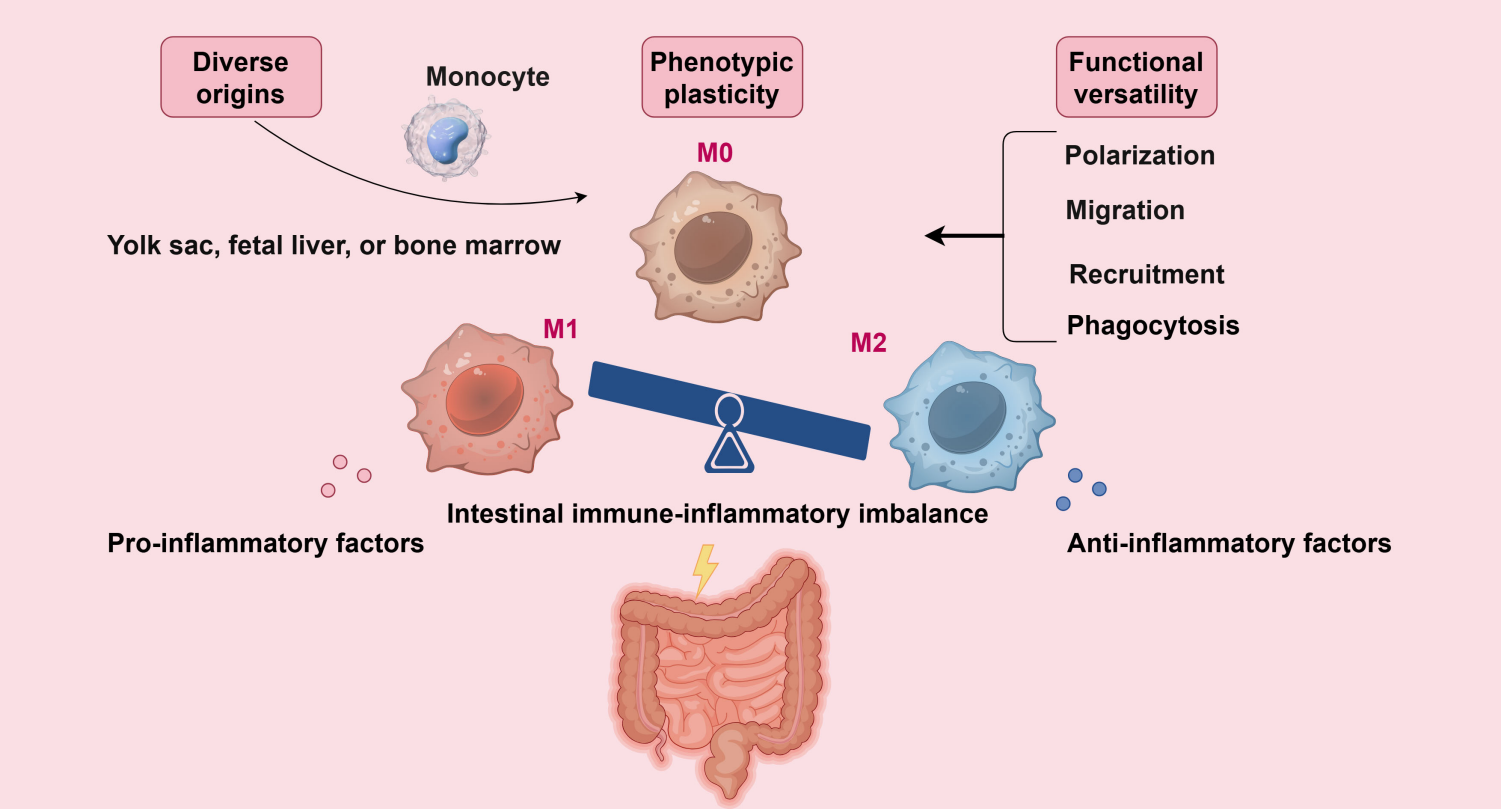
Macrophages originate from various sources, exhibit phenotypic plasticity, and display functional diversity, making them crucial regulators of intestinal inflammation development and progression (By Figdraw).

An imbalance in macrophage polarization—particularly excessive M1 activation during disease flares and impaired M2-driven resolution during remission—has been implicated in the pathogenesis of multiple inflammatory disorders (41–43). Given the reversible nature of macrophage polarization, targeting their functional state represents a promising therapeutic strategy for inflammatory diseases, notably UC (26, 36) (**Figure 1**).

## 3 The dual role of macrophages in UC

### 3.1 Pro-inflammatory effects: driving intestinal inflammation and barrier disruption

Macrophages exhibit pronounced pro-inflammatory properties in the pathogenesis of UC. Upon intestinal barrier damage, pathogenic bacteria or damage-associated molecular patterns activate lamina propria macrophages via pattern recognition receptors, triggering their polarization toward the M1 phenotype. Activated M1-like macrophages (inflammatory macrophages) subsequently secrete pro-inflammatory cytokines such as tumor necrosis factor-alpha (TNF), interleukin-6 (IL-6), and interleukin-1β (IL-1β), which are critical for pathogen clearance but also exacerbate intestinal inflammation and mucosal barrier injury in UC. Spalinger et al. demonstrated that macrophage-specific PTPN2 deficiency promotes IL-6 upregulation, reducing the expression of tight junction proteins (Claudin-2, occludin) and increasing colonic permeability in mice, thereby elevating the risk of inflammatory bowel disease (IBD) (44). Intriguingly, IL-6 inhibition reversed these barrier defects and inflammatory infiltration (45), confirming the detrimental role of M1 macrophage-derived cytokines in UC progression. Further studies reveal that the toll-like receptor 2/4 (TLR2/4)-NF-κB-NOD-like receptor thermal protein domain associated protein 3 (NLRP3) axis exacerbates colitis by inducing macrophage pyroptosis and IL-1β secretion, amplifying intestinal inflammation and barrier dysfunction (46). Single-cell RNA sequencing highlights that UC patient-derived macrophages display M1-polarized signatures, with elevated CXCL9 (T-cell chemoattractant) and CD40 (T-cell activation marker), intensifying Th1/Th17-mediated immunity (47). Additionally, Tim-3–deficient macrophages recruit neutrophils and induce necroptosis, disrupting the mucosal barrier and perpetuating a vicious cycle of colitis (48).

### 3.2 Reparative functions: guardians of homeostasis and tissue repair

Despite their pro-inflammatory dominance in UC, macrophages also play indispensable reparative roles via M2 polarization. During inflammation resolution, macrophages transition to an M2-like phenotype (anti-inflammatory macrophages), secreting arginase-1 (Arg1) and interleukin-10 (IL-10) to promote epithelial regeneration and extracellular matrix remodeling (49–51). Notably, Arg1 from M2 macrophages provides polyamine precursors for collagen synthesis, facilitating tissue repair (52, 53). Numerous studies have demonstrated that various signaling pathways mediated by M2 macrophages reduce colonic inflammation and enhance the repair of the intestinal barrier. For instance, the activation of the M2-type macrophage/Foxo3 axis has been shown to alleviate the colonic inflammatory response and improve intestinal mucosal barrier function in mice with dextran sulfate sodium (DSS)-induced colitis (54). Additionally, the activation of the M2 macrophage/Wnt/ERK signaling pathway can significantly aid in the repair of the intestinal mucus barrier during colitis (55). Notably, the protective effects of therapeutic drugs on colitis were significantly diminished after the depletion of macrophages (56). It is believed that inhibiting macrophage apoptosis may be a crucial strategy for slowing down the progression of UC (57). Additionally, many chemokines and cytokines produced due to M2 macrophage polarization help restore the Th1/Th17 balance, which significantly alleviates colitis (58–60).

In conclusion, Macrophages in UC are a double-edged sword—both drivers of inflammation and mediators of repair. Deciphering their regulatory mechanisms unlocks therapies to amplify beneficial functions while mitigating harm, offering hope for UC treatment.

## 4 Regulation and operational mechanisms of macrophage function in UC

### 4.1 Metabolic reprogramming

#### 4.1.1 Metabolic reprogramming and macrophage plasticity

Macrophages exhibit an extraordinary ability to adapt, known as high plasticity, which enables them to adjust their phenotypic characteristics and functions in response to subtle changes in their microenvironment. This remarkable plasticity is primarily regulated by the cell’s metabolic state (61). Metabolic reprogramming is a key process through which cells actively modify their metabolic pathways to adapt to various physiological and pathological conditions. In macrophages, metabolic reprogramming plays a crucial role in determining their activation state and effector functions; it is a core mechanism in the processes of macrophage polarization and function regulation (61). By finely regulating glucose, lipid, and amino acid metabolism, metabolic reprogramming impacts not only the energy supply of macrophages but also the efficiency of signaling and gene expression patterns (62–64). This comprehensive regulation ultimately influences the phenotypic traits and functional capabilities of macrophages. For example, an excess of classical M1 macrophages combined with a deficiency of alternative M2 macrophages can lead to severe colitis, a condition that can be significantly reversed through the modulation of metabolic reprogramming. This presents a highly innovative and practical approach for the clinical treatment of UC.

#### 4.1.2 Role of macrophage metabolic reprogramming in colitis

Glycolysis is a metabolic pathway that converts glucose into pyruvate and lactate through a series of enzyme-mediated reactions in the cytoplasm (65). Upregulation of glycolytic rates enhances the pro-inflammatory properties of macrophages activated by pathogens (66). Numerous studies have indicated that increased glycolysis in macrophages can augment their pro-inflammatory characteristics and contribute to the development of various inflammatory diseases (67–69). The classical M1 polarization program primarily relies on glycolysis and is regulated by key glycolytic enzymes such as pyruvate kinase M2 (PKM2) and 6-phosphofructo-2-kinase/fructose-2,6-biphosphatase 3(PFKFB3) (66). In experiments involving colitis in mice, the expression of PFKFB3 was significantly upregulated, which coincided with an increase in glycolytic activity and a higher ratio of M1 macrophages (70). Notably, these effects were reversed and colonic inflammation was alleviated following the administration of the PFKFB3-specific inhibitor 3PO (70). HIF-1α is highly expressed in macrophages stimulated by LPS and IFN-γ, functioning as a key transcription factor that regulates the expression of glycolytic enzymes (71–73). The combined activation of LPS and IFN-γ appears to stabilize HIF-1α subunits and induce metabolic reprogramming of M1 macrophages towards glycolysis (74). Recent studies have demonstrated that the ubiquitination of HIF-1α inhibits glycolysis and promotes a balance between M1 and M2 macrophages, which could improve conditions like UC (75). PKM2 activators, such as DASA-58 and TEPP-46, can directly inhibit the expression of M1 macrophages induced by LPS while enhancing the expression of several marker proteins associated with M2 macrophages. When LPS promotes the expression of PKM2, HIF-1α, and IL-1β in macrophages, these components form a complex that inhibits the increase of glycolysis in macrophages and reduces the expression of pro-inflammatory signals (76). Further research has indicated that the dimerization of PKM2 is crucial for maintaining the stability of the HIF-1α protein; this dimerization enhances glycolysis and leads to the polarization of macrophages towards a pro-inflammatory phenotype in colitis models, which can be detrimental to recovery from colitis (77, 78). These studies have highlighted the crucial role of key enzymes in the glycolytic pathway in regulating the proportion of inflammatory macrophages and alleviating colitis. They provide a theoretical foundation for further exploration of how these enzymes impact inflammatory macrophage regulation and the management of colitis. This research not only enhances our understanding of the interactions between metabolism and immunity in the development of UC, but also suggests a potential direction for developing novel therapeutic strategies that target the glycolytic pathway. In addition, mTORC1 signaling induces macrophages to adopt a pro-inflammatory state and plays a crucial role in the development of various inflammatory diseases (79–81). Recent studies have shown that regulating glycolysis and M1 polarization facilitate the recovery from colitis by inhibiting the mTORC1/HIF-1α signaling pathway; this therapeutic effect can be counteracted by the mTORC1 agonist, l-leucine (82). IL-10 is a well-known anti-inflammatory cytokine that contributes to the treatment of many inflammatory diseases (83, 84). Research by Eddie et al. has indicated that IL-10 reduces colonic inflammation in mice with colitis by inhibiting lipopolysaccharide-induced glucose uptake and glycolysis in macrophages while promoting OXPHOS; furthermore, in macrophages from colitis mice and patients with IBD, a deficiency of IL-10 exacerbates mitochondrial damage and enhances the activation of mTORC1 signaling, leading to increased expression of NLRP3 and IL-1β (85). These studies not only highlight the role of mTORC1 signaling pathway in the process of macrophage metabolic reprogramming and polarization, but also clarify the mechanism of IL-10 as its key upstream regulator. Together, these findings create a multi-layered network that links metabolic regulation, signaling, and immune responses. This framework offers a strong theoretical foundation and significant opportunities for further research aimed at developing new therapeutic strategies for UC and other chronic inflammatory conditions. Interestingly, recent findings have revealed that M2 macrophages can also promote glycolysis, which challenges the traditional belief that glycolysis is minimal in anti-inflammatory macrophages (86). However, reducing glycolysis has not affected the activation of M2 macrophages (87). Overall, enhanced glycolysis in macrophages contributes to the development of UC. Therefore, manipulating the expression of M1 macrophages through the regulation of glycolytic reprogramming remains an important strategy for promoting recovery from colitis.

The cessation of tricarboxylic acid(TCA) cycle metabolism is another feature of M-type 1 macrophages and is closely linked to macrophage-mediated inflammatory and immune responses (88). Research has demonstrated that succinate, a byproduct of TCA cycle metabolism, accumulates at sites of inflammation and modulates immune function through signaling via the succinate receptor (SUCNR1); this receptor signaling has been shown to influence immune responses, potentially leading to intestinal inflammation and compromised intestinal barrier function (88). Patients with UC and mice with DSS-induced colitis exhibit increased expression of SUCNR1, while SUCNR1-silenced mice show significantly greater resistance to DSS-induced colitis (89). Recent studies have also found that succinate exacerbates colonic injury and the inflammatory response in DSS-induced colitis models by inhibiting the expression of Treg cells, which have anti-inflammatory properties (90). These studies reveal the important regulatory role of metabolic arrest in the TCA cycle and its key intermediate, succinate, in macrophage-mediated intestinal inflammation. When this metabolism ceases, it prevents the next steps in processing succinic acid, leading to the progression and persistence of colonic inflammation. Targeting the accumulation of succinic acid or its downstream signaling pathways offers a new strategy for treating UC.

Lipogenesis involves a series of enzymatic reactions that synthesize fatty acids and triglycerides. The metabolism of fatty acids is a significant pathway for generating pro-inflammatory effects in M1 macrophages (91). M1 macrophages synthesize fatty acids and utilize them as precursors for inflammatory mediators while primarily obtaining most of their adenosine triphosphate (ATP) from aerobic glycolysis (92). In contrast, M2 macrophages rely on a functional mitochondrial respiratory chain driven by fatty acid oxidation (FAO) to fuel the TCA cycle and subsequent OXPHOS (92). FAO is a catabolic process that breaks down fatty acids into acetyl-CoA, which is then used in the TCA cycle and OXPHOS (93). Although FAO is not exclusive to anti-inflammatory macrophages and is also present in M1 macrophages, this phenomenon does not appear to impact the pro-inflammatory effects of M1 (92, 94, 95). Numerous studies have shown that FAO in macrophages is crucial for influencing their anti-inflammatory effects (96–98). Peroxisome proliferator-activated receptors (PPARs) are a class of nuclear receptors and transcription factors that regulate gene expression; they play key roles in various processes, including lipid metabolism, energy balance, inflammatory response, and cell differentiation (99–101). In the context of FAO, the activation of PPARs affects fatty acid uptake, transport, and oxidation processes, acting as a regulatory switch in fatty acid management (102). Free fatty acid receptors (FFARs) sense changes in the concentration of intracellular and extracellular free fatty acids and regulate the rate of FAO through signaling pathways (103, 104). Research by Li et al. involving DSS-induced mice and LPS-induced RAW264.7 cells treated with the FFAR4 agonist GSK137647 found that the FFAR4-PPARα axis regulates FAO and promotes M2 macrophage polarization (105). This suggests that the FFAR4-PPARα axis may play a role in modulating M2 macrophage polarization, alleviating colitis (105). Similar studies have indicated that the symptoms of UC, colon length, and tissue damage in DSS-induced colitis mice were significantly improved through the combined intervention of the FFAR1 agonist GW9508 and the FFAR4 agonist GSK137647 (106). Moreover, the *in vitro* experiments of this study indicated that these improvements in colitis symptoms and histopathology have been achieved by promoting FAO, which reduces lipid accumulation and enhances M2 macrophage polarization (106). Recently, it was reported that mTORC2/PPAR-γ-mediated increases in FAO could induce M2 polarization, thereby improving colonic tissue inflammation and alleviating symptoms in colitis mice (82). These studies demonstrate the critical role of fatty acid metabolism in the polarization of macrophages and their inflammatory responses. Regulating fatty acid metabolism in macrophages can not only shift their polarization but also significantly aid in the resolution of tissue inflammation. This approach offers a promising therapeutic avenue for the treatment of UC.

The reprogramming of amino acid metabolism in macrophages also plays a significant role in the development of UC. Specifically, the regulation of amino acid metabolism directly influence the response mechanisms of macrophages; this metabolic adaptation not only supports the functional activities of macrophages but also plays a crucial role in their development, ensuring the stability of their polarization state within specific microenvironments (107). Traditionally, two metabolites of amino acids, iNOS and Arg1, are recognized as characteristic markers of anti-inflammatory and pro-inflammatory macrophages, respectively (108). This method of distinguishing macrophage phenotypes based on amino acid markers shows that the metabolic processes associated with different macrophage phenotypes are distinct, highlighting their important relationship with inflammation. Chen et al. discovered that tryptophan catabolism products significantly accumulate during the activation of M1 macrophages, as determined through metabolite set enrichment analysis (86). Tryptophan metabolism serves as a primary means of distinguishing between M1 and M2 macrophages. This study also indicated that using dual inhibitors of tryptophan catabolic enzymes enhance the expression of anti-inflammatory factors in macrophages (86). The ability to promote the expression of anti-inflammatory factors through these dual inhibitors exemplifies a typical process for reprogramming macrophages to switch their phenotype. Lu et al. demonstrated that branched-chain amino acid (BCAA) metabolite-mediated alterations in the TCA cycle modulate M2 macrophage polarization in response to specific immune responses and inflammatory conditions (109). They achieved this by inducing M2 polarization in mice using chitin and by knocking down genes for key enzymes involved in BCAA metabolism in M2 macrophages *in vitro (* 109). Additionally, the metabolism of other amino acids, such as L-glutamine, serine, and glycine, influence the phenotype of both macrophage types by affecting the TCA cycle; this modulation subsequently impacts the inflammatory response and immune regulation of macrophages (108, 110, 111). These studies underscore the critical role of amino acid metabolic reprogramming in determining the pro- or anti-inflammatory properties of macrophages. In human primary macrophages, branched-chain aminotransferase 1 (BCAT1) is the dominant isoform of BCAT while the specific inhibitor of it is ERG240 (112). Papathanassiu et al. found that the expression of BCAT1 influences the polarization of macrophages in mice with various inflammatory diseases; by using an ERG240 inhibitor in model mice with multiple inflammatory conditions, they suggested that BCAT1 may play a role in the development of these diseases and thus regulate their progression (112). Chen et al. employed several functional enrichment algorithms to analyze tryptophan metabolism and activation patterns in different cell types in UC; they discovered that the tryptophan metabolism in macrophages plays a regulatory role in the abnormal immune response and inflammation observed in UC (21). A recent study indicated that 5-methoxytryptophan reduces M1 polarization of macrophages and alleviates colonic inflammation in mice with DSS-induced colitis (113). This findings suggest that the catabolism of 5-methoxytryptophan is crucial for transforming the macrophage phenotype in colitis mice. Hashimoto et al. reported that serum levels of D-alanine were significantly lower in patients with UC compared to healthy controls; they found that the transformation of inflammatory macrophages was inhibited, and colitis symptoms in mice were alleviated through intraperitoneal injection of D-alanine into DSS-induced colitis mice (114). The metabolic changes of D-alanine in colitis mice contribute to the remission of colitis. Zhang et al. studied macrophage-specific knockout of ring finger protein 99 (RNF99) in DSS-induced colitis mice and examined RNF99-overexpressing inflammatory macrophages *in vitro*; they demonstrated that RNF99 degrades TAK1-binding protein 2 (TAB2) through the lysine ubiquitination pathway, thereby regulating the inflammatory state of macrophages and promoting recovery from colitis (115). This research highlights the significance of lysine metabolic reprogramming in regulating macrophage polarization and its role in colitis development.

**Figure 2:**
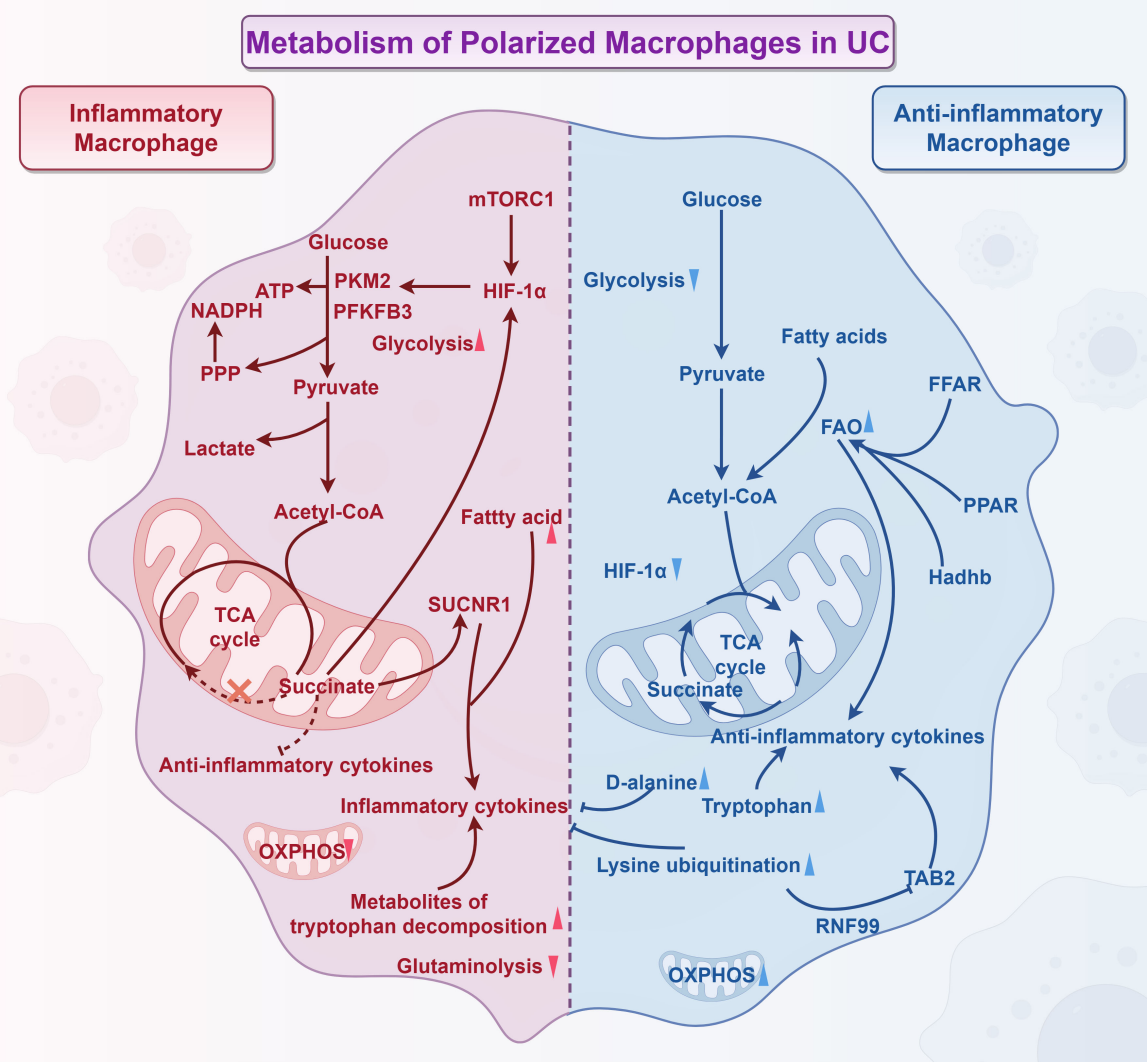
Metabolism of polarized macrophages in UC (By Figdraw). Inflammatory macrophage: 1) The classical polarization of inflammatory macrophages primarily relies on glycolysis, which is regulated by key enzymes such as PKM2 and PFKFB3. 2) IL-10 serves as a crucial upstream regulator of mTORC1-mediated glycolysis. The mTORC1/HIF-1α signaling pathway influences glycolysis and M1 polarization, promoting the recovery from colitis. 3) Succinate, a byproduct of TCA cycle metabolism, accumulates at sites of inflammation and regulates immune function through SUCNR1 signaling. This accumulation leads to intestinal inflammation and damages the intestinal barrier. Succinate also modulates HIF-1α expression, which indirectly affects macrophage glycolysis levels. Furthermore, succinate inhibits the expression of regulatory T cells, which have anti-inflammatory properties, thereby exacerbating intestinal inflammation. 4) Fatty acids serve as precursors for the synthesis of inflammatory mediators in M1 macrophages. 5) The accumulation of tryptophan metabolites, along with decreased L-glutamine catabolism, encourages macrophages to transition to the M1 phenotype. Anti-inflammatory macrophage: Anti-inflammatory macrophages utilize FAO to drive the functional mitochondrial respiratory chain, which powers the TCA cycle and subsequent OXPHOS. FAO plays a crucial role in enhancing the anti-inflammatory effects of M2 macrophages. The expression of proteins such as FFAR, PPAR, and Hadhb all promote FAO, contributing to the improvement of colitis. Additionally, the ubiquitination of lysine along with the accumulation of D-alanine and tryptophan regulate macrophage polarization and reduce inflammation in macrophages.

In summary, cellular metabolism serves as a key regulatory factor in macrophage polarization and functional modulation, and targeting these metabolic pathways may represent a promising strategy for modulating macrophages to treat colitis. Future studies should focus on optimizing such metabolic regulatory approaches and translating them into clinical applications, while maintaining a solid theoretical foundation to achieve more effective inflammation control and disease management (**Figure 2**).

### 4.2 Beyond metabolism

#### 4.2.1 Macrophage epigenetic regulation and colitis

Cellular epigenetics refers to the chemical modifications that regulate gene expression without altering the DNA sequence (116). These modifications impact gene expression by changing the structure of DNA and proteins on chromosomes, and they are both heritable and reversible (117, 118). The biological characteristics of macrophages are closely linked to their epigenetic regulatory networks. The epigenetic regulation mechanisms in macrophages involve various molecular modifications, including DNA methylation, histone post-translational modifications, and the regulation of non-coding RNAs; these epigenetic factors play a significant role in regulating macrophage phenotypic plasticity (119). Research conducted by Solier et al. identified a chemically active pool of copper ions in the mitochondria of inflammatory macrophages, which regulates the redox cycle of NAD by catalyzing the activation of hydrogen peroxide; this unique metabolic mechanism involving metal ions drives the macrophage epigenetic regulatory network toward a pro-inflammatory phenotype by maintaining intracellular NAD dynamics (120). Furthermore, studies have shown that the histone H3K9 acetylation and H3K18 lactate modifications promote a shift of M1 macrophages towards an anti-inflammatory phenotype (121, 122). It is important to note that the role of lactate—as a product of macrophage glycolysis—and glycolysis itself in the inflammatory response is complex and multifaceted. There exists a mutually reinforcing relationship between these processes, as well as a balance mechanism of mutual inhibition. This intricate interaction is essential for the body’s ability to maintain homeostasis and adaptability in response to pathophysiological challenges such as inflammation. Histone methyltransferases are a class of enzymes that catalyze the transfer of methyl groups to histones; these modifications are crucial for regulating gene expression (123). Histone H3 is a core histone in chromatin, which, along with other histones such as H2A, H2B, and H4, forms the basic structure of chromatin (124–126). SET1 is a histone methyltransferase that specifically methylates lysine 4 on histone H3 (127). Megakaryoblastic Leukemia 1 (MKL1) influences cellular behavior by recruiting SET1 to the promoter region of P65 target genes, thereby initiating pro-inflammatory transcription in macrophages (128). An et al. found that mice deficient in MKL1 were less sensitive to DSS-induced colitis; they observed that macrophages from transgenic mice expressing human MKL1 were more sensitive to DSS-induced colitis than those from normal control mice (129). In these transgenic mice, the number of macrophages in the lamina propria was decreased, and most of them exhibited a pro-inflammatory phenotype (129). The regulatory effect of MKL1 on macrophage phenotype in colitis could be attributed to epigenetic modifications mediated by MKL1, which require further experimental validation. Jumonji domain-containing 3 (JMJD3) is an important histone demethylase that regulates epigenetic modifications in macrophages, thus influencing macrophage phenotypes (130). It has been implicated in various immune-inflammatory diseases (130–132). Recent study has shown that the inhibition the JMJD3-NLRP3 signaling axis alter the trimethylation of histone H3 on lysine 27 (H3K27me3) in macrophages, which alleviates the colonic inflammatory state in DSS-induced colitis mice (133). In a study conducted by Duan et al., the JMJD3-specific inhibitor GSK-J4 was used to intervene in mice with DSS-induced colitis and in inflammatory macrophages. The researchers found that this intervention significantly improved the inflammatory state and alleviated colonic pathological changes. These enhancements resulted from the JMJD3 manipulation of H3K27me3 in macrophages, which led to a higher proportion of anti-inflammatory macrophages and a decreased expression of pro-inflammatory factors (133). Epigenetic modifications in macrophages play a crucial role in regulating their phenotypic responses, offering novel insights into the pathogenesis of UC. Targeting key enzymes involved in these epigenetic processes has been shown to influence macrophage polarization, dampen excessive inflammatory activity, and support tissue homeostasis. A deeper understanding of how these modifications shape macrophage function may guide the identification of more precise therapeutic targets. Further investigation into the underlying mechanisms could therefore contribute to the development of innovative interventions for UC.

#### 4.2.2 Chemokine-mediated macrophage response and colitis

Chemokines, also called chemotactic cytokines, are small signaling proteins secreted by cells (134). They are named for their ability to induce directional chemotaxis, guiding the movement of nearby responding cells (135, 136). During the inflammatory response, chemokines play a crucial role by directing the migration and proliferation of inflammatory cells, including macrophages; this participation is vital for chemotaxis, cell aggregation, and the activation of various immune cells, which can significantly contribute to the development of inflammatory diseases and the rapid formation of an immune-inflammatory response (18). Chemerin is a chemotactic protein that plays an essential role in the inflammatory response by recruiting macrophages to the site of inflammation, primarily through its interaction with the macrophage-expressed chemerin receptor 1 (CMKLR1) (137). A study by Dander et al. on DSS-induced colitis in CMKLR1 knockout mice found that the deletion of this chemokine receptor increased the mice’s sensitivity to DSS, resulting in more severe colonic pathology (138). This research highlights the importance of immune cell surface chemokine activity in the development of colitis. Additionally, it has been reported that the chemokine PC3-secreted microprotein (PSMP) is expressed in the colon tissues of patients with colitis (139). Moreover, the expression of this chemokine in colon tissues was found to be up-regulated in the early stages of DSS-induced colitis (139). Researchers discovered that intervening in mice with DSS-induced colitis using adenoviral methods or PSMP-neutralizing antibodies resulted in PSMP promoting the expression of multiple inflammatory factors during the early stages of colitis, furthermore, this intervention prompted the conversion of macrophage phenotypes into pro-inflammatory macrophages (139). Xue et al. conducted a study on DSS-induced colitis in mice and LPS-induced inflammation in RAW264.7 cells, finding that reducing the migration of macrophages induced by C-C motif chemokine ligand 2 (CCL2), specifically by decreasing both migration distance and velocity, can help alleviate colitis (140). Chemokines produced by macrophages also play a significant role in influencing colitis by impacting other immune cells. Research conducted by Jones et al. on patients with IBD and mice with DSS-induced colitis demonstrated that macrophages producing the chemokines C-C motif chemokine ligand 7 and C-C motif chemokine ligand 8 recruit more monocytes from the bloodstream into colon tissue; these recruited monocytes serve as a major source of IL-1β and TNF in the inflamed tissue (19). This study reveals a harmful cycle of inflammation in colonic disorders:monocytes are attracted to the site of inflammation, where they release pro-inflammatory factors that worsen the condition; at the same time, macrophages release chemokines to draw in more monocytes, further perpetuating and intensifying the inflammation. Overall, chemokines are closely linked with macrophages as small-molecular-weight cytokines or signaling proteins. Regulating these chemokines could be a strategic approach to limit UC by promoting the migration of immune cells, including macrophages, to areas of colonic inflammation.

#### 4.2.3 Microbial metabolism-mediated macrophage polarization and colitis

Gut microbiota and their metabolites play a crucial role in regulating the immune system (141). By understanding how microbial metabolism products affect the differentiation and function of immune cells, we can gain insights into the pathogenesis of various systemic diseases and develop new strategies for their prevention and treatment. Macrophages are crucial immune cells that sense environmental changes and play a role in regulating immunity of various diseases (142, 143). The gut microbiota influences macrophage characteristics through microbial signals, and this interaction is crucial in the development of various inflammatory conditions, particularly UC (144–146). Short-chain fatty acids (SCFAs) are organic fatty acids containing 1 to 6 carbon atoms, and they are the main end products of anaerobic bacteria in the colon (147). In patients with UC, there is a significantly low abundance of anaerobic bacteria that produce SCFAs (54). Research has demonstrated that a mixture of supernatants containing seven SCFAs promotes an anti-inflammatory macrophage phenotype in the colon by inhibiting the Janus Kinase-Forkhead box O3 signaling pathway, which leads to improved intestinal inflammation and enhanced barrier function in mice with DSS-induced colitis (54). Butyrate, one of the SCFAs produced by gut microbes during metabolism, has also been studied for its effects on macrophages. A recent study using clodronate liposomes to deplete macrophages found that butyrate increases mucus production and the proportion of mucus-secreting goblet cells in the colonic crypts in a macrophage-dependent manner (55). Researchers demonstrated both *in vivo* and *in vitro* that butyrate stimulates the polarization of macrophages toward the M2 phenotype, which, in turn, promotes goblet cell formation and aids in repairing the mucus barrier, especially following DSS-induced injury (55). Butyrate has protective effects in UC by promoting the polarization of macrophages toward an anti-inflammatory phenotype, enhancing goblet cell function, and aiding in mucus barrier repair. This finding uncovers a novel interaction mechanism between macrophages and goblet cells and suggests that butyrate is a potential therapeutic target for UC. Interestingly, individuals with gastrointestinal motility disorders are more prone to developing colonic inflammation (148). The accumulation of linoleic acid caused by intestinal flora imbalance in patients with gastrointestinal motility disorder leads to the recruitment of pro-inflammatory macrophages, which is susceptible to colitis (149). Additionally, polyamines produced by the gut microbiota, such as putrescine and spermidine, play a vital role in maintaining intestinal health (150, 151). These polyamines enhance the proliferation of colonic epithelial cells, regulate macrophage differentiation, and alleviate colitis symptoms (152). It has also been reported that putrescine produced by bacteria acts as a substrate for symbiotic metabolism and increases the number of anti-inflammatory macrophages in the colon to help alleviate colonic tissue inflammation and maintain intestinal mucosal homeostasis (152). These findings highlight the crucial role of microbial metabolism in mediating macrophage responses that support host intestinal health and offer new perspectives for treating intestinal inflammation.

#### 4.2.4 Macrophage apoptosis and autophagy in colitis

Apoptosis refers to the process of cell autonomous and orderly death controlled by genes to maintain the stability of the internal environment (44). It has complex molecular biological mechanisms and important biological significance, it plays an important role in the growth and development of organisms, the stability of the internal environment and the resistance to external factors (153). The disorder of macrophage apoptosis process is closely related to the occurrence of a variety of inflammation-related diseases (154, 155). Therefore, it is of great significance to study the mechanism and regulation of apoptosis for the treatment and prevention of diseases. In addition to regulating macrophage polarization and chemotaxis, inhibiting macrophage apoptosis is another strategy for managing UC. Cai et al. conducted a study on mice with DSS-induced colitis and inflammatory macrophages; they found that intervention with exosomes that highly express miR-378a-5p inhibits the activation of the NLRP3 inflammasome and the secretion of IL-18 and IL-1β (57). Additionally, this intervention reduced the cleavage of Caspase-1, thereby inhibiting the occurrence of pyroptosis and alleviating DSS-induced colitis (57). This discovery offers new insights and potential drug targets for treating IBD. Liu et al. conducted a study on mice with colitis induced by DSS and on bone marrow-derived macrophage models triggered by LPS; they discovered that salidroside inhibits the activation of the NLRP3 inflammasome by blocking the triggering receptor expressed on myeloid cells 1; this inhibition leads to a reduction in macrophage pyroptosis and decreases the release of the inflammatory factor IL-1β (156). This study highlights the promising potential of drugs designed to improve colitis by regulating macrophage autophagy. Farnesoid X receptor (FXR) is a nuclear receptor, mainly expressed in the liver and intestine, involved in the regulation of bile acid metabolism, inflammatory response and intestinal barrier function, and its activation has anti-inflammatory effects (157, 158). Recently, Yang et al. found that the supernatant from saccharomyces boulardii (SB) activates FXR, thus inhibiting NLRP3 expression and preventing macrophage apoptosis in DSS-induced colitis mice, which significantly improves the symptoms of colitis. Additionally, *in vitro* experiments showed that the effect of SB is reversed by the FXR inhibitor Guggulsterone, which increases levels of apoptosis-related proteins (159). This underscores the importance of macrophage apoptosis in protecting against colitis. While SB is beneficial, other strains worsen UC by regulating autophagy, with the specific mechanisms still under investigation. In Shen et al.’s study, peptostreptococcus anaerobius aggravated DSS-induced colitis in mice by inducing macrophage pyroptosis and IL-1β secretion through the activation of the TLR2/4-NF-κB-NLRP3 signaling axis (46). These studies illustrate that the distorted macrophage apoptotic response is one of the intrinsic mechanisms of intestinal dysbiosis leading to colitis in patients with UC.

Overall, in UC, defective and insufficient apoptosis prolongs and amplifies the inflammatory response. Meanwhile, the role of autophagy in the pathogenesis of UC has attracted considerable attention in recent years. Impaired autophagy in macrophages compromises their ability to clear pathogens and regulate inflammation, thereby exacerbating the disease (160, 161). Autophagy is a crucial mechanism for maintaining cellular homeostasis, playing an important role in immune regulation in inflammatory bowel disease by degrading damaged organelles, misfolded proteins, and intracellular pathogens (162). In the context of UC, dysfunctional autophagy resulting from genetic polymorphisms in macrophage autophagy-related genes is closely associated with disease onset and progression (163). Altered expression of autophagy-related genes can impair the formation of autophagosomes, thereby reducing the capacity of macrophages to eliminate invasive Escherichia coli; this leads to persistent intestinal inflammation and increases susceptibility to chronic inflammatory responses (164, 165).

At the molecular level, macrophage autophagy contributes to the pathology of UC through multiple mechanisms (161). Autophagy serves as a key regulator of the NLRP3 inflammasome. An intact autophagic pathway facilitates the clearance of inflammasome activators such as damaged mitochondria, suppresses caspase-1 activation, and reduces the production of pro-inflammatory cytokines including IL-1β and IL-18, thereby preventing excessive inflammation (166). Conversely, impaired autophagy leads to hyperactivation of the NLRP3 inflammasome and aggravates intestinal inflammation. Besides, there is a dynamic interaction between macrophage autophagy and the gut microbiota (167). On one hand, the intestinal microbial community regulates macrophage autophagy activity via metabolites and pattern recognition receptor signaling; on the other hand, macrophages help maintain microbial homeostasis and eliminate invading pathogens through autophagy (168–170). In UC, this balance is disrupted, creating a vicious cycle of dysbiosis and autophagy dysfunction that drives disease progression.

Notably, macrophage autophagy is also involved in UC pathogenesis through the modulation of immune responses. It influences antigen presentation and helps maintain immune homeostasis by removing inflammatory signaling molecules, such as reactive oxygen species (ROS) and inflammasome components (171–173). Macrophages with defective autophagy exhibit increased production of pro-inflammatory cytokines and impaired anti-inflammatory functions, promoting a chronic inflammatory state in UC (161).

**Figure 3:**
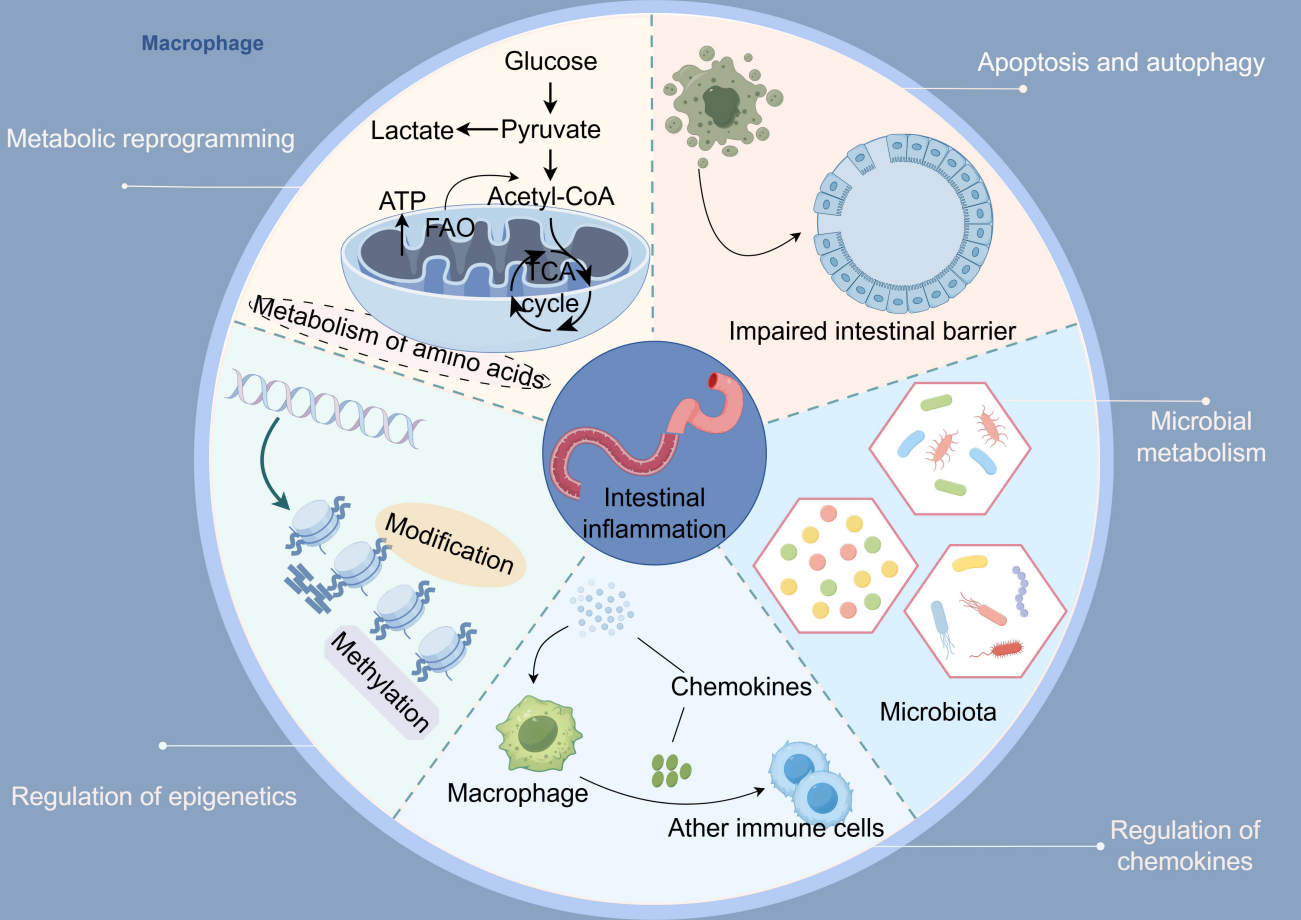
Macrophages regulate five key axes involved in the pathogenesis of ulcerative colitis: 1) metabolic reprogramming, 2) epigenetics, 3) chemokine networks, 4) apoptosis and autophagy, and 5) microbes along with their metabolites (By Figdraw).

Abnormalities in macrophage apoptosis and autophagy silently contribute to the initiation and progression of UC. Further investigation into the specific mechanisms and targets underlying dysregulated macrophage apoptosis and autophagy represents a promising therapeutic strategy for colitis. Incorporating the regulation of macrophage apoptosis and autophagy into UC treatment research enhances conventional immunomodulatory approaches. Moreover, this exploration offers new perspectives for the future development of precision immunotherapies focused on modulating cell fate (**Figure 3**).

## 5 Targeting macrophages for UC therapy

Intestinal inflammation and immune imbalance represent core pathogenic mechanisms underlying the onset and progression of UC, with macrophages serving as pivotal mediators in this complex interplay. As key innate immune cells residing in the intestinal lamina propria, macrophages exhibit remarkable functional plasticity, dynamically switching between pro-inflammatory and anti-inflammatory phenotypes in response to environmental cues. Dysregulated macrophage activation contributes significantly to mucosal damage, epithelial barrier disruption, and persistent immune activation in UC. Consequently, modulating macrophage polarization, infiltration, and cytokine production has emerged as a promising therapeutic strategy. Recent advances in immunology and pharmacology have led to the identification of multiple targeted agents that regulate macrophage function in UC.

These include:

**Nanoformulations:** Engineered nanoparticles capable of delivering drugs specifically to macrophages, thereby enhancing therapeutic efficacy while minimizing systemic side effects.
**Natural or Synthetic Compounds:** Compounds such as a-aminobutyric acid (AABA), xylan acetate (XylA), and genistein that modulate macrophage signaling to improve the balance of macrophage polarization.
**Microbial Preparations:**Probiotics, specifically Akkermansia muciniphila (A. muciniphila) and Lactobacillus johnsonii (L. johnsonii), have an impact on macrophage behavior through interactions between the host and the microbes.
**Extracts and compounds of traditional Chinese medicine (TCM):** Extracts and compounds of TCM that exert anti-inflammatory effects by regulating macrophage polarization and cytokine secretion.

These strategies offer novel insights into precision immunomodulation in UC and highlight the potential of macrophage-targeted therapies to restore immune homeostasis and promote mucosal healing (**Table 1**).

**Table 1:** Summary of potential targeted macrophages therapeutics for UC.

Category	Drug name	Model	Mechanism of action	Refs
Nanoformulations	HA-Cr nanogel	DSS-induced colitis mice	IL-1β↓,TNF↓,IL-6↓,iNOS↓,Arg1↑,IL-10↑	(180)
		LPS-stimulated RAW264.7 cells	IL-1β↓,TNF↓,IL-6↓,iNOS↓,Arg1↑,IL-10↑	
	Se-CA	DSS-induced colitis mice	IL-1β↓,TNF↓,IL-6↓,CD86↓,phenylalanine↓,tyrosine↓,tryptophan↓,CD206↑	(11)
		LPS-induced RAW264.7 cells	IL-1β↓,TNF↓,IL-6↓,ROS↓,CD86↓,CD206↑	
	RMN NPs	DSS-induced colitis mice	IL-1β↓,TNF↓,IL-6↓,IL-10↑,CD86↓,iNOS↓,CD206↑,Arg-1↑,IL-1R↑,Fizz-1↑	(184)
Natural or Synthetic Compounds		LPS-induced RAW264.7 cells	IL-1β↓,TNF↓,IL-6↓,IL-10↑,CD86↓,iNOS↓,CD206↑,Arg-1↑	
	AABA	DSS-induced colitis mice	Nos2↓,TNF↓,IL-6↓,iNOS↓,CD80↓,CD86↓	(187)
		LPS-activated BMDM cells	Nos2↓,TNF↓,IL-6↓,iNOS↓,ECAR↓,H3K27me3↑	
	XylA	DSS-induced colitis mice	LPS↓,TLR4↓,MD2↓,MyD88,IL-1β↓,TNF↓,IL-6↓,CD86↓	(191)
		LPS-induced RAW264.7 cells	TNF↓,IL-6↓,NO↓,CD86↓,SCFAs↑	
	Genistein	DSS-induced colitis mice	IL-1β↓,TNF↓,LPS↓,IL-6↓,iNOS↓,IL-10↑,Arg-1↑	(195)
		DSS-induced colitis mice	TNF↓,IL-6↓,iNOS↓,IL-10↑,Arg-1↑	
Microbial Preparations	A. muciniphila	DSS-induced colitis mice	IL-1β↓,TNF↓,IL-6↓,HDAC5↓,iNOS↓,Arg-1↑	(204)
		LPS-induced RAW264.7 cells	TNF↓,IL-6↓,iNOS↓,CD86↓,CD206↑,Arg-1↑,IL-10↑	
	L.johnsonii GLJ001	LPS/IFN-γ-induced THP-1 cells	IL-1β↓,TNF↓,iNOS↓,CD86↓,Gpr41↓,Gpr43↓	(206)
Extracts and Compounds of TCM	FN	DSS-induced colitis mice	IL-1β↓,TNF↓,CD86↓,iNOS↓,Arg1↑,IL-10↑	(210)
		LPS-induced RAW264.7 cells	IL-6↓,iNOS↓,MHC-II↓,CD86↓,IL-10↑,Arg-1↑	
	Bilobalide	DSS-induced colitis mice	IL-1β↓,TNF↓,IL-6↓,p-p65↓	(213)
		LPS/IFN-γ-induced BMDM cells	IL-1β↓,TNF↓,IL-6↓,MHC-II↓,CD11c↓,p-p65↓	
	STV-Na	DSS-induced colitis mice	IL-1β↓,TNF↓,NF-κB p65↓,CD86↓,CD206↑	(214)
	coptisine	DSS-induced colitis mice	IL-1↓,TNF↓,IL-6↓,CD86↓,iNOS↓,p-ERK1/2↓,p-4-EBP1↓,CD206↑,IL-10↑,IL-4↑,Arg1↑,TSC1↑	(217)
		LPS/IFN-γ-induced RAW264.7 cells	IL-12↓,TNF↓,iNOS↓,p-ERK1/2↓,p-4-EBP1↓,IL-10↑,TGF-β↑,Arg1↑,TSC1↑,m6A↑,Mettl14↑	
Compounds of TCM	TXYF	DSS-induced colitis mice	IL-1β↓,TNF↓,IL-6↓,CD86↓,CD206↑,IL-10↑,IL-4↑	(220)
		LPS/IFN-γ/ATP -induced BMDM cells	CD86↓,iNOS↓,IL-1β↓,p-p65↓,TNF↓,IL-6↓,NLRP3↓,CD206↑,IL-10↑,IL-4↑	
	CSD	DSS-induced colitis mice	iNOS↓,cGAS↓,Arg1↑,IL-10↑	(224)
		Interferon stimulated DNA-induced RAW264.7 cells	TNF↓,iNOS↓,cGAS↓,Arg1↑,IL-10↑	
	CDD-2103	DSS-induced colitis mice	TNF↓,F4/80+↓	(225)
		LPS/IFN-γ-induced BMDM cells	CCL2↓,p-p38 MAPK↓,CD86↓,iNOS↓	

### 5.1 Nanoformulations

Nanoformulations have demonstrated significant potential in the treatment of UC. They utilize nanocarriers, such as liposomes and polymer nanoparticles, to achieve targeted delivery and controlled release of drugs (174). This approach enhances the local concentration of medications in the inflamed intestine, improve drug delivery efficiency, and reduce systemic exposure and side effects (174, 175). The advantages of nanoformulations include enhanced drug stability, improved bioavailability, prolonged retention time of the drug, and the ability to actively target inflammatory sites through surface modification (176–178). These features help regulate the intestinal immune-inflammatory response, making nanoformulations an effective and precise strategy for UC therapy. Creatine (Cr), a nitrogen-containing organic acid that provides energy to cells, has shown potential benefits in treating UC. Research indicates that oral administration of Cr helps prevent the development of the disease (179). Huai et al. combined Cr with nanomaterials to create a Cr-modified selenium-based hyaluronic acid nanogel, referred to as HA-Cr Nanogel; this formulation exploits the CD44 targeting ability of hyaluronic acid and its positive charge at the site of inflammation for precise delivery (180). The researchers found that HA-Cr nanogel was not only more effective than Cr alone but also more specifically targeted inflammation in mice with DSS-induced colitis and in Raw 264.7 cells (180). Recently, Li et al. used chitosan (CS) as the backbone and covalently modified L-arginine (L-Arg) onto CS through an EDC/NHS catalytic system to form a polymeric complex known as CS-L-Arg (CA); they then prepared a modified selenium nanozyme, referred to as Se-CA, through a chemical reduction method (11). In both *in vitro* and *in vivo* studies, they validated the anti-UC properties of Se-CA, finding that Se-CA significantly improves colonic pathology in colitis mice by enhancing epithelial repair, reducing inflammation, reversing macrophage type 1 responses, and decreasing reactive oxygen species (ROS) levels (11). Importantly, long-term use of Se-CA was found safe (11). Additionally, the PPAR gamma agonist Rosiglitazone (RLZ) is believed to have therapeutic effects in various inflammatory diseases by inducing macrophages to switch to the M2 phenotype and secrete multiple anti-inflammatory factors (181–183). Similar studies have utilized nanoparticles coated with macrophage membranes (from RAW264.7 cells) and loaded with Rosiglitazone (RLZ) form RMN NPs; these nanoparticles effectively modulate the M1 to M2 macrophage ratio and protect intestinal epithelial cells *in vitro*; *in vivo*, they significantly improve pathological changes in the colon, reduce levels of inflammatory factors in DSS mice, and restore the intestinal barrier function (184). Compared to nanoparticles or free drugs alone, RMN NPs specifically target inflamed intestinal tissue, producing superior therapeutic effects at the lesion site (184). In conclusion, although these nanomedicines show promise in treating UC, their potential application is still unclear. Further research and improvements are needed regarding large-scale production, long-term safety, targeting efficiency, individualized treatment, and stability. There is a new expectation for better utilization of nanomaterials to regulate macrophage function in colon tissue for the treatment of this refractory disease.

### 5.2 Natural or synthetic compounds

AABA is a non-protein amino acid that can be metabolized from various neutral amino acids, previously thought to be linked to metabolic sexual dysfunction (185, 186). Li et al. proposed that AABA regulates macrophage polarization and function in UC, providing a new foundation for targeting amino acid metabolism to modulate macrophage function for UC treatment (187). The researchers examined DSS-induced colitis in mice and LPS-induced inflammatory macrophages, findings that AABA reduces disease severity in colitis-affected mice through metabolic reprogramming and epigenetic modification of M1 macrophages (187). Specifically, AABA enhances the amino acid metabolism in macrophages, increases H3K27 trimethylation in the promoter region of M1 macrophage-associated inflammatory genes, and inhibits the glycolytic process of cells (187). These results suggest that AABA plays a crucial role in macrophage development, significantly reducing inflammation and excessive immune responses in the colon. Xylan and its derivatives, as insoluble polysaccharides that are difficult to digest in the stomach and duodenum after oral administration, play a crucial role in re-establishing intestinal homeostasis (188). For instance, xylan butyrate ester and wheat bran arabinoxylan are potent compounds that help regulate the balance between intestinal inflammation and immunity, promoting recovery from UC (189, 190). Interestingly, a recent study by Tang et al. demonstrated that XylA, a derivative of xylan, significantly alleviates the symptoms and pathological manifestations of UC; they found that in both DSS-induced colitis in mice and LPS-induced inflammation in RAW264 cells, XylA significantly reduces the polarization of M1 macrophages in the inflammatory colon and decreases the expression of various pro-inflammatory factors in an *in vitro* inflammatory environment (191). In addition, these effects are closely linked to the short-chain fatty acids (SCFAs) derived from XylA. Genistein is a significant active compound derived from soybean, known for its wide range of biological functions, including regulating the cell cycle, anti-inflammatory effects, antioxidant properties, and promoting autophagy (192). These attributes make it valuable in treating various diseases, such as diabetes, lipid disorders, and acute pancreatitis (193, 194). Recently, Jia et al. demonstrated that Genisteind enhances symptom recovery and improves colonic histomorphology in mice with DSS-induced colitis (195). It was also found to reduce the level of inflammatory cell infiltration in the affected colon (195). This therapeutic effect primarily stems from Genistein’s ability to regulate macrophage phenotypes, leading to a decrease in pro-inflammatory macrophages and an increase in anti-inflammatory macrophages in diseased mice. Consequently, Genistein is regarded as a promising macrophage-targeted therapeutic agent for UC.

### 5.3 Microbial preparations

Intestinal epithelial cells are constantly exposed to a complex microenvironment composed of various immune cells and immeasurable microorganisms, which has a tremendous impact on the normal function of the intestinal barrier (196). Multiple studies have shown that microbial agents and their metabolites are able to modulate beneficial effects on the host by improving gut microbial balance, enhancing intestinal barrier function, and improving local inflammatory-immune responses (197, 198). Unfortunately, in recent years, there have not been many studies on the treatment of UC using microbial agents that target macrophages. Given the importance of macrophage polarization balance in the pathogenesis of UC, it is important to continue to explore the mechanism of various microbial agents-mediated macrophage treatment of UC. A. muciniphila regulates macrophage polarization state to participate in the occurrence and development of various diseases including hepatocellular carcinoma and intestinal infectious diseases (199, 200). Histone deacetylase 5 (HDAC5) belongs to the HDAC family and is able to alter chromatin structure and function by removing acetyl groups on histone lysine residues, thereby repressing gene transcription (201). It can also interact with specific transcription factors and be recruited to specific gene promoter regions to achieve fine regulation of specific gene expression (202). Recently, it was reported that the abundance of A.muciniphila is significantly decreased in UC, which may be related to the development of UC (203). Miao et al. demonstrated through their study of DSS-induced colitis in mice and inflammatory macrophages that A. muciniphila can reduce the expression of HDAC5; this reduction prevents the H3K9 acetylation modification of the promoter for the HDAC5 deacetylation-disabled homolog 2 gene, resulting in the suppression of this gene’s expression no longer occurring (204). Consequently, this mechanism leads to a reduction in pro-inflammatory macrophage polarization in the colonic tissue. The study delves deeper into the role of A. muciniphila-mediated epigenetic modification in the inflammatory phenotype of macrophages in UC and enriches the existing targets for macrophage phenotype regulation, which has contributed to the expansion of potential macrophage-targeting drugs for UC. Jia et al. conducted a study on L. johnsonii to explore its relationship with pathogenicity in mice with DSS-induced colitis (205). Both *in vivo* and *in vitro* studies demonstrated that L. johnsonii promotes an increase in the ratio of intestinal M2 macrophages by activating the STAT3 signaling pathway; this process enhances the expression of anti-inflammatory factors, effectively controlling intestinal inflammation (205). Recently, Cai et al. demonstrated that L. johnsonii GLJ001, which becomes enriched in rat feces following intragastric administration of Pu-erh tea, plays a crucial role in restoring the macrophage-mediated inflammatory-immune balance in colitis (206). Specifically, their research, involving DSS-induced colitis in mice and inflammatory THP-1 cells, revealed that L. johnsonii GLJ001 influences the levels of SCFAs, which are closely linked to macrophage polarization, by improving gut microbiota balance in colitis-affected mice (206) Furthermore, the use of SCFAs to intervene in an *in vitro* inflammatory macrophage model reduces inflammation and significantly decreases the levels of GPR41 and GPR43, which play a role in immune and inflammatory responses (206). Therefore, L. johnsonii GLJ001 presents as a potential therapeutic agent to promote recovery from UC by modulating macrophage polarization through the gut microbiota/SCFAs axis.

### 5.4 Extracts and compounds of TCM

Formononetin (FN) is an isoflavone that plays a significant role in maintaining the balance between inflammation and immunity, and it is the primary active compound found in Pueraria montana and astragalus (207–209). A study by Xiao et al. demonstrated that FN improves symptoms, reduces colonic lesions, and decreases mortality in mice with DSS-induced colitis (209). Importantly, both *in vivo* and *in vitro* studies have shown that FN alleviates UC by promoting the transformation of inflammatory colonic tissues from pro-inflammatory to anti-inflammatory macrophages; this transformation may be crucial in the pathogenesis of UC (210). However, the efficacy of FN can be diminished by the depletion of macrophages using clodronate liposomes (210). Bilobalide is a natural active ingredient extracted from ginkgo biloba leaves and is widely researched for its potential in treating neurological disorders and inflammatory diseases (211, 212). Zhang et al. discovered that bilobalide significantly improves colon shortening and damage while reducing inflammation in mice with DSS-induced colitis (212). *In vitro* studies on inflammatory macrophages showed that bilobalide decreases the expression of inflammatory phenotype markers such as MHC-II and CD11c, as well as multiple inflammatory factors produced by these macrophages; this therapeutic effect is attributed to the modulation of the NF-κB pathway (213). A similar study involving mice with DSS-induced colitis highlighted isosteviol sodium (STV-Na), a terpenoid sodium salt derived from the acid hydrolysis of stevioside; this compound may also ameliorate UC by inhibiting NF-κB p65 signaling, thereby reducing M1 macrophage polarization (214). Various TCM extracts have shown promise in alleviating colonic inflammation and improving symptoms of UC through NF-κB/p65 signaling-mediated M1 macrophage polarization and this research provides valuable insights for further exploring the mechanisms of other TCM extracts that have therapeutic effects on UC. Coptisine is an active ingredient extracted from Coptis chinensis, known for its ability to treat various inflammatory diseases, including IBD and psoriasis (215, 216). A recent study has shown that Coptisine regulates the epigenetics of macrophages, which helps reduce the number of inflammatory macrophages in the colon, ultimately aiding in the treatment of UC (217). Researchers conducted experiments on mice with DSS-induced colitis and found that Coptisine improves colonic pathology and symptoms while altering the polarization balance of macrophages in the colonic tissue to enhance the anti-inflammatory response (217). Further *in vivo* and *in vitro* studies revealed that Coptisine regulates the ERK pathways by methylating TSC1, which mediates the polarization balance of macrophages and contributes to the improvement of UC (217). Epigenetics involves modifications that change the expression of target proteins without altering the gene sequence. Therefore, changing the epigenetics of macrophages is a significant method for regulating their function. In summary, the outstanding potential of TCM extracts in regulating macrophage function for UC treatment is highlighted.

### 5.5 Compounds of TCM

Compounds of TCM are composed of various herbs that work together in a coordinated manner, following the hierarchical roles of the sovereign, the minister, the assistant, and the courier within the theoretical framework of TCM. Each herb in the formulation interacts with the others to achieve therapeutic effects that may be difficult to attain with a single herb alone. Tongxie-yaofang (TXYF) is a safe and effective compound of TCM used for treating UC, although its precise therapeutic mechanism remains unclear (218, 219). Research conducted by Zhang et al. demonstrated that TXYF restores the polarization balance of M1 and M2 macrophages, while also improving both the pathological changes in colon tissues and the symptoms of UC in mice with colitis (220). Furthermore, their study found that TXYF reverses inflammation induced by LPS and IFN-γ in macrophages *in vitro*, a process linked to the NF-κB-mediated activation of NLRP3 (220). This research provides valuable insights into the therapeutic mechanism of TXYF and offers a theoretical basis for the clinical application of TXYF-targeted macrophage therapy in the treatment of UC. Compound sophorae decoction (CSD) is commonly used to treat UC, particularly in improving patients’ blood stool conditions (221). Many studies have demonstrated that CSD intervenes in the occurrence and progression of UC through multiple targets and pathways (222, 223). However, there are few studies specifically addressing its effects on macrophages. Wu et al. discovered that, in DSS-induced colitis mice, the expression of pro-inflammatory factors TNF and IL-6 was significantly down-regulated, while the level of the anti-inflammatory factor IL-10 was significantly up-regulated in colon tissue (221). These changes may result from phenotypic shifts in macrophages. They further supported this hypothesis by examining macrophage phenotype changes in the spleen and mesenteric lymph nodes of the mice. Unfortunately, they did not conclusively prove whether the changes in inflammatory factors were solely due to the shifts in macrophage phenotype. Fei et al. recently confirmed that CSD targets macrophages in the treatment of UC using a combination of *in vivo* and *in vitro* experiments along with a network pharmacology approach (224). Specifically, they identified hundreds of targets associated with CSD that have therapeutic effects on UC, including proteins such as inducible nitric oxide synthase (iNOS) and TNF, which are classic products of macrophage polarization (224). Subsequently, *in vivo* and *in vitro* experiments confirmed that CSD alleviated UC via cyclic GMP-AMP synthase (cGAS)-mediated macrophage polarization (224). This study enhances our understanding of CSD’s role in treating UC and contributes to the mechanistic research concerning its modulation of macrophage function. A similar study used the theories of traditional Chinese medicine to develop a new decoction called modified Zhenwu decoction (CDD-2103), it targets macrophage function and migration through the C-C Chemokine receptor 2/p38 mitogen-activated protein kinase (p38MAPK) signaling axis, which is involved in the regulation of macrophage migration, however, adoptive macrophage transfer experiments undermined the protective effect of this treatment on colitis (225). This further confirms that CDD-2103 primarily treats UC by targeting macrophage function and behavioral changes.

All of the above are the latest therapeutic drugs with the potential to target macrophages. Focusing on macrophages represents a promising new approach for managing UC. The role and behavior of macrophages are crucial in the onset and progression of UC. Therefore, it remains a significant topic within the digestive health community to develop methods for precisely regulating the types of macrophages that aid in UC recovery and effectively directing drug treatment to the affected areas of the colon.

## 6 Conclusions

Currently, there is no consensus on the pathogenesis of UC, but the primary pathological feature is the imbalance of intestinal inflammation and immune response. Metabolic reprogramming signals in macrophages and signals beyond metabolism collectively alter macrophage function and behavior, significantly influencing intestinal inflammation and modifying local inflammatory-immune dysregulation in the gut. Ultimately, this helps in the treatment of UC. Therefore, targeting the functions and behaviors of macrophages to improve intestinal inflammation is a crucial strategy in UC treatment. This review provides a theoretical basis for macrophage-targeted therapy in UC and offers important new avenues for future research.

## 7 Future perspectives

Future therapeutic directions can also further explore single-cell omics appge co-culture models, and nanotechnology-enabled targeted delivery systems, aiming to bridge mechanistic understanding with clinical translation for precision medicine in UC management. Despite promising anti-inflammatory effects observed with macrophage-targeted therapies in preclinical models, their successful clinical translation remains limited by several practical challenges. One of the most clinically relevant issues is the phenomenon of “loss of response (LoR)”, wherein patients who initially respond well to treatment gradually lose efficacy over time—a problem frequently encountered with biologic agents such as anti-TNF monoclonal antibodies (226). LoR is distinct from primary non-response and is often attributed to multiple interrelated mechanisms, including the development of anti-drug antibodies, activation of compensatory pro-inflammatory pathways, and microbial-driven alterations in drug metabolism or target expression (227, 228). Moreover, adaptive changes within the host immune system, such as shifts in T-cell subsets or altered macrophage polarization dynamics, may further compromise therapeutic durability (81, 229). To address these challenges, emerging strategies include therapeutic drug monitoring to guide dose optimization and combination therapy with immunomodulators, such as azathioprine, to reduce immunogenicity (230, 231). Nonetheless, more mechanistic studies and well-designed clinical trials are warranted to fully understand the underlying biology of treatment failure and to develop rational interventions that improve long-term outcomes for patients with UC.

This review primarily focuses on the regulatory roles of macrophages in the pathogenesis of UC while also exploring potential therapeutic interventions targeting these cells. It is essential to recognize the long-term oncogenic risks linked to chronic intestinal inflammation. Patients with long-term UC have an increased susceptibility to colitis-associated cancer (CAC), a complication driven by persistent immune dysregulation and epithelial damage. In this context, tumor-associated macrophages (TAMs) play a crucial role in tumor initiation and progression. They contribute to significant processes such as remodeling the immune microenvironment, promoting angiogenesis, and facilitating immune evasion (232, 233). Remarkably, TAMs exhibit considerable plasticity and undergo functional changes when transitioning from chronic inflammation to malignancy. This transition involves a shift from a pro-inflammatory M1-like phenotype to an immunosuppressive M2-like phenotype (234). Accompanying this phenotypic switch are alterations in metabolic programming—such as enhanced lipid metabolism and downregulated tryptophan metabolism—along with the activation of signaling pathways like the phosphorylation of STAT6 and protein kinase B (235–237). Targeted inhibition of these changes that promote colitis-associated cancer could theoretically enhance human health. Specifically, a deeper understanding of these dynamic changes may inform the development of novel therapeutic strategies aimed at modulating macrophage function to prevent or delay the onset of CAC. Future studies should concentrate on defining the temporal evolution of macrophage subsets and their interactions with tumor cells and other stromal components throughout the inflammatory-to-malignant transition in UC.

## Abbreviations

TNF: tumor necrosis factor
IL-6: interleukin-6
IL-1β: interleukin-1β
IBD: inflammatory bowel disease
DSS: dextran sulfate sodium
Arg1: arginase-1
IL-10: interleukin-10
IL-4: interleukin-4
PKM2: pyruvate kinase M2
PFKFB3: 6-phosphofructo-2-kinase/fructose-2,6-biphosphatase 3
NLRP3: NOD-like receptor thermal protein domain associated protein 3
ROS: oxygen species
TCA: tricarboxylic acid
ATP: adenosine triphosphate
FAO: fatty acid oxidation
PPAR: speroxisome proliferator-activated receptors
FFARs: free fatty acid receptors
Hadhb: Hydroxyacyl Coenzyme A Dehydrogenase Beta
BCAA: branched-chain amino acid
BCAT1: branched-chain aminotransferase 1
RNF99: ring finger protein 99
MKL1: megakaryoblastic leukemia 1
JMJD3: jumonji domain-containing 3
H3K27me3: histone H3 on lysine 27
CMKLR1: chemerin receptor 1
PSMP: PC3-secreted microprotein
CCL2: C-C motif chemokine ligand 2
SCFAs: short-chain fatty acids
FXR: farnesoid X receptor
SB: saccharomyces boulardii
TCM: traditional Chinese medicine
Cr: creatine
CS: chitosan
L-Arg: L-arginine
CA: CS-L-Arg
RLZ: rosiglitazone
AABA: a-aminobutyric acid
XylA: xylan acetate
A. muciniphila: Akkermansia muciniphila
HDAC5: histone deacetylase 5
L. johnsonii: lactobacillus johnsonii
FN: formononetin
STV-Na: isosteviol sodium
TXYF: Tongxie-yaofang
CSD: compound sophorae decoction
iNOS: inducible nitric oxide synthase
TAB2: TAK1-binding protein 2
OXPHOS: oxidative phosphorylation
ECAR: extracellular acidification rate
IL-1R: interleukin-1R
Fizz-1: found in inflammatory zone
TLR4: toll-like receptor 4
MD2: myeloid differentiation protein 2
p-p65: phospho-NF-κB p65
p65: NF-κB p65
p-ERK1/2: phosphorylated extracellular regulated protein kinases 1/2
p-4-EBP1: phosphorylated 4E binding protein 1
TSC1: tuberous sclerosis complex 1
m6A: N6-methyladenosine
Mettl14: methyltransferase-like protein 14
cGAS: cyclic GMP-AMP synthase
p-p38 MAPK: phospho-P38 mitogen-activated proteinkinase
CAC: colitis-associated cancer
TAMs: tumor-associated macrophages
LoR: loss of response.
